# Impact of Submerged Fermentation Parameters on Proteins Extracted from *Ganoderma sichuanense* and Their Antioxidant Potential

**DOI:** 10.3390/microorganisms14010133

**Published:** 2026-01-07

**Authors:** Vítor Alves Pessoa, Larissa Ramos Chevreuil, Roziane Rodrigues Nunes, Daiane Barão Pereira, Giovanna Lima-Silva, Larissa Batista do Nascimento Soares, Aldenora dos Santos Vasconcelos, Sérgio Dantas de Oliveira-Junior, Walter J. Martínez-Burgos, Ceci Sales-Campos

**Affiliations:** 1 Postgraduate Program in Biotechnology, Universidade Federal do Amazonas, Av. General Rodrigo Octavio, Manaus 69067-005, Amazonas, Brazil; barao.dbp@gmail.com (D.B.P.); giovannalimafr@gmail.com (G.L.-S.); ceci.cog@gmail.com (C.S.-C.); 2 Edible Fungi Cultivation Laboratory, Instituto Nacional de Pesquisas da Amazônia, Av. André Araújo, Manaus 69067-375, Amazonas, Brazil; larissachevreuil@gmail.com (L.R.C.); rozirodnunes@gmail.com (R.R.N.); lbbnascimento@gmail.com (L.B.d.N.S.); aldenora.svasconcelos@gmail.com (A.d.S.V.); sergiodantas100@hotmail.com (S.D.d.O.-J.); 3 Postgraduate Program in Biodiversity and Biotechnology of the Bionorte Network, Universidade do Estado do Amazonas, Av. Carvalho Leal, Manaus 69065-001, Amazonas, Brazil; 4 Centro Multiusuário para Análise de Fenômenos Biomédicos, Universidade do Estado do Amazonas, Av. Carvalho Leal, Manaus 69065-001, Amazonas, Brazil

**Keywords:** lingzhi, bioprocess, polypeptides, ultrastructure, secondary structure, oxidative stress, cytoprotective, metabolism modulation

## Abstract

*Ganoderma sichuanense* is a widely studied medicinal mushroom, but the production of its antioxidant proteins has been scarcely evaluated. We assess the influence of different concentrations of culture media components under submerged fermentation, with and without agitation, on production of proteins with antioxidant activity from the mycelial biomass of *G*. *sichuanense*. Protein extracts were characterized by scanning electron microscopy, X-ray diffraction, and attenuated total reflectance Fourier-transform infrared spectroscopy. They were also analyzed for total protein and phenolic contents, antioxidant activities (ABTS^•+^, DPPH^•^, chelating ability, and reducing power), and electrophoretic profiles by SDS-PAGE. The most active extract was tested for cytoprotective potential under H_2_O_2_-induced oxidative stress in *Saccharomyces cerevisiae*. Growth kinetics of the best fermentation condition were also analyzed. Microstructural differences ranged from fibrillar to aggregated forms, depending on cultivation. Crystallinity was unaffected, but chemical differences and secondary structure organization were confirmed by infrared spectroscopy. The extract from the static culture with 10 g·L^−1^ glucose, 5 g·L^−1^ yeast extract, and 2.5 g·L^−1^ soy peptone (referred as CM1S) showed the highest protein and phenolic contents and the strongest antioxidant activity (IC_50_ = 4.8 and 24.0 µg of protein·mL^−1^ for ABTS^•+^ and DPPH^•^, respectively). SDS-PAGE revealed higher protein band intensities in static cultures. CM1S showed potential to protect yeast cells from oxidative stress. The Gompertz model estimated a specific growth rate of 0.0068 h^−1^ in CM1S. The findings highlight a cultivation strategy that modulates fungal metabolism and improves the recovery of antioxidant proteins from *G*. *sichuanense* biomass.

## 1. Introduction

Among natural antioxidants, several small molecules are recognized. However, macromolecules such as proteins and their derivatives can also exhibit notable antioxidant potential, depending on their amino acid composition and conformational structure [1]. Antioxidant proteins not only resist damage caused by free radicals but also protect cellular components against oxidative stress, acting as allies against a range of pathological conditions [2].

A Fungal Immunomodulatory Protein (FIP) synthesized by the mushroom *Ganoderma lucidum* (FIP-glu or LZ-8) has demonstrated significant antioxidant potential. Additionally, it promotes keratinocyte proliferation, inhibits melanoma cell development, and suppresses melanin synthesis. When compared to vitamin C this protein has been highlighted as a potential ingredient in products aimed at tissue repair and skin lightening [3]. In cellular models, FIP-glu also exhibited immunomodulatory capacity, activating macrophages (RAW264.7) and displaying both proinflammatory and anti-inflammatory activity [4].

FIPs have been described in several *Ganoderma* species, including *G*. *lucidum*, *G*. *tsugae*, *G*. *japonicum*, *G*. *microsporum*, *G*. *applanatum*, *G*. *atrum*, *G*. *sinense*, *G*. *bonisense*, *G*. *amboinense*, *G*. *tenus*, and *G*. *capense*. These proteins are composed of 106 to 133 amino acids, with molecular weight around 12 kDa, and are rich in aspartic acid (Asp) and valine (Val) residues, with low levels of histidine (His), cysteine (Cys), and methionine (Met). They also exhibit high homology and four different conserved amino acid fragments (WGRG, DKAYTYRV, SDGSQK, and AQWN), suggesting evolutionary conservation within the *Ganoderma* genus [5]. However, the content of FIPs in mushrooms is relatively low, with 5 to 10 mg of FIP-glu per 300 g of *G*. *lucidum* mycelium [6].

Other protein derivatives from *G*. *lucidum* have also been reported with antioxidant activity, including a selenium-enriched protein (Se-GL-P) [7], the antioxidant enzymes catalase (CAT) and superoxide dismutase (SOD) [8], crude protein extracts [9], a proteoglycan [10], and a glycopeptide [11]. Thus, other polypeptides synthesized by *Ganoderma* may also have great antioxidant activity with potential for use in health-beneficial formulations.

*Ganoderma sichuanense* is a mushroom whose medicinal properties are widely recognized in Asian countries, making it a species of significant ecological and commercial importance. Studies have confirmed its potential as an anti-tumor, antiviral, anti-aging, hypoglycemic, hypocholesterolemic, hepatoprotective, hypotensive, and antioxidant agent [12,13]. This species has encountered various taxonomic issues, often being mistakenly referred to as *G*. *lucidum* in several publications and incorrectly identify as *G*. *lingzhi* [14]. Recent articles suggest that, given current knowledge, publications should prioritize the name *G*. *sichuanense* [15].

Furthermore, our research group has already described the potential of the CC22 strain of *G*. *sichuanense* in the synthesis of proteins, both in basidioma from solid fermentation [16], and mycelial biomass from submerged fermentation [17], including the protein class of proteases inhibitors, which has already been reported to present antioxidant activity. For example, a protease inhibitor from the mushroom *Agaricus bisporus* (AbPI) exhibited high antioxidant and protective activity in a yeast-induced oxidative stress model, as evidenced by the maintenance of cellular integrity [18]. In cellular models using adipocytes (3T3-L1), AbPI was also able to reduce the formation of intracellular reactive oxygen species (ROS) and increase glucose uptake, demonstrating an effect comparable to that of commercial drugs. This suggests AbPI as a potential candidate for use as an oral therapeutic agent in the management of oxidative stress and diabetes [19].

The production of protein concentrates enriched with different antioxidant proteins derived from the *Ganoderma* species represents a promising alternative for use as active ingredients in cosmetics, medicines and protein supplementation, however strategies to enhance production in submerged fermentation are required. Therefore, we aimed to evaluate the influence of culture media component concentrations and agitation on the production of antioxidant proteins from *G*. *sichuanense* in submerged fermentation, as well as to assess how these conditions affect the structural properties of the recovered proteins. Given the lack of studies addressing these aspects in *G*. *sichuanense*, this investigation helps to fill an important knowledge gap in medicinal mushroom biotechnology and bioprocess development.

## 2. Materials and Methods

### 2.1. Biological Material and Submerged Fermentation

The *G*. *sichuanense* CC22 (=*Ganoderma lingzhi*) was accessed from the Collection of Cultures of Microorganism of Silvicultural Interest at the Instituto Nacional de Pesquisas da Amazônia (INPA). The strain was maintained in PDA medium (potato dextrose agar) and then cultivated in different proportions of culture media (CM) (Table 1), at pH 6.0, 25 °C, with agitation at 120 rpm (A) or static (S), for 12 days. Media formulations and cultivation conditions followed a previous study by our group [17], which identified glucose, yeast extract, and soy peptone as key components.

### 2.2. Protein Obtention and Quantification

The mycelial biomass was homogenized in 0.15 M sodium chloride (1:20, m/v) for 2 h at 120 rpm and 10 °C. Afterward, the material was centrifuged for 20 min at 10,000× *g* and 4 °C. The supernatant was dialyzed in cellulose membranes with a 12 kDa cut-off against distilled water for 48 h under refrigeration (4 °C), yielding protein fractions of medium to high molecular mass. It was then centrifuged again for 20 min at 4.667× *g* and 4 °C, followed by lyophilization [17]. Protein content in the extracts was determined using Bradford method [20]. The total phenolic compounds (TPC) content was estimated using the Folin–Ciocalteu reagent and gallic acid as the standard, with the results expressed in micrograms of Gallic Acid Equivalence (GAE) [21].

### 2.3. Structural Analysis of Protein Extracted

The protein extracts were characterized by Scanning Electron Microscopy (SEM) using a Phillips XL-30ESEM electron microscope (Philips Electron Optics, Eindhoven, The Netherlands), with a 15 kV electron beam and a magnification of 100×. X-ray Diffraction (XRD) analysis was performed using a Shimadzu XRD-6000 spectrometer (Shimadzu Corporation, Kyoto, Japan), with copper Kα radiation, a voltage of 30 kV, and a current of 15 mA, at a scan rate of 2.0° per minute over a 2θ continuous range from 4.0° to 70.0°.

The chemical groups present in the protein extracts were investigated by Attenuated Total Reflectance Fourier-Transform Infrared Spectroscopy (ATR-FTIR). For this, 5 mg of each sample were analyzed using a Cary 630 spectrometer (Agilent Technologies, Santa Clara, CA, USA), in the range of 400 to 4000 cm^−1^, with a resolution of 8 scans. The characterization of the secondary protein structure in the samples was carried out using the amide I band region (1700–1600 cm^−1^), through Fourier self-deconvolution and Gaussian fitting. The percentages of secondary structures were calculated by integrating and combining the fitted curves, using RStudio (version 2025.09.2) .

### 2.4. Evaluation of Antioxidant Activity

The antioxidant activity of the protein extracts was evaluated using ABTS^•+^ and DPPH^•^ inhibition assays, ferrous ion chelating ability, and reducing power of ferric ion, employing methodologies adapted for 96-well microplates [22]. For testing, the protein extracts were initially resuspended in distilled water at 10 mg·mL^−1^. When a sample showed inhibition above 50% in ABTS^•+^ and DPPH^•^ assays, a serial dilution (10, 5, 2.5, 1.25, 0.625 and 0.3125 mg·mL^−1^) was performed to calculate the amount that inhibits 50% of the radical (IC_50_). In the reducing power assay, the increase in the absorbance indicated higher antioxidant potential of the samples.

### 2.5. Sodium Dodecyl Sulfate–Polyacrylamide Gel Electrophoresis (SDS-PAGE)

The protein extracts were subjected to sodium dodecyl sulfate—polyacrylamide gel electrophoresis (SDS-PAGE) [23]. Molecular weight markers (Promega, Madison, WI, USA) ranging from 10 to 225 kDa were employed to estimate the molecular weight of proteins present in the samples.

### 2.6. Cytoprotective Effect

The protein extract with the highest antioxidant potential was analyzed for cytoprotective activity in an oxidative stress-induced model of *Saccharomyces cerevisiae* (MTCC 1972; Florax^®^, Hebron Farmacêutica, São Paulo, Brazil). The yeast cells were cultured for 6 h in YPD medium (yeast peptone dextrose), pH 6.0, at 200 rpm and 28 °C. The cells were harvested, washed twice with autoclaved distilled water, and resuspended in phosphate-buffered saline (pH 7.4). The suspension was standardized to an absorbance of 0.323 at 660 nm (~4 × 10^6^ cells) and incubated with 2 mM hydrogen peroxide and the protein extract (1 mg·mL^−1^) at 37 °C for 1 h [18]. After treatment, the cells were prepared [24] and imaged using a scanning electron microscope (SEM—Jeol JSM IT500HR, JEOL Ltd., Tokyo, Japan) with an acceleration voltage of 5 kV in secondary electron mode. A control was performed without the protein extract.

### 2.7. Fungal Growth Kinetics

The experiment condition of submerged fermentation with the highest antioxidant activity of protein extract was subjected to fungal growth kinetics assays. *G*. *sichuanense* was cultivated in a medium containing glucose (10 g·L^−1^), soy peptone (2.5 g·L^−1^), and yeast extract (5 g·L^−1^) without agitation. Three Erlenmeyers flasks were taken daily for 12 days to evaluate biomass production parameters (dried biomass in g·L^−1^) and specific growth rate (h^−1^). The fermented broth was used to monitor pH and glucose consumption (reducing sugars in g·L^−1^ by 3,5-dinitrosalicylic acid (DNS) method) [17].

### 2.8. Experimental Design and Statistical Analysis

A complete factorial design in a 2^3^ scheme was applied to evaluate the effects of three factors: glucose (X_1_), peptone (X_2_), and agitation (X_3_), each at two levels (low: −1 and high: +1), with soluble protein concentration as the response variable. A total of eight experimental conditions were defined based on this design (Table 2). These conditions were used to assess the impact of the cultivation factors on protein production during submerged fermentation, and the resulting data were analyzed using RStudio. Statistical analyses from quantification assays were performed using Statistica 7 software, with data from triplicates subjected to analysis of variance (ANOVA). Means were compared by Tukey’s test with a significance level of 1% (*p* < 0.01). IC_50_ values were calculated using non-linear regression in inhibitor vs. normalized response—variable slope method, and specific growth rates were estimated by mathematical models fitting the data in non-linear regression, both using the software GraphPad Prism 9.4. Principal Component Analysis (PCA) was used for statistical comparison of the chemical profile of each protein extract by FTIR-ATR, using OriginPro 2025.

## 3. Results

### 3.1. Soluble Protein and Total Phenolic Compounds (TPC)

**Figure 1:**
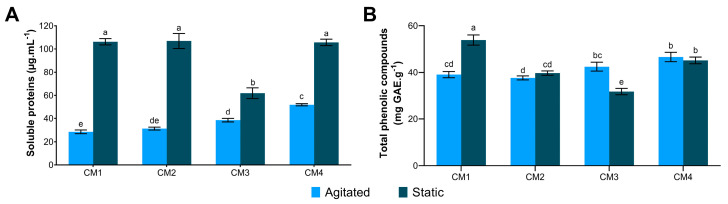
Quantification of molecules in protein extracts of *G*. *sichuanense* cultivated under different submerged fermentation conditions. (**A**) Soluble protein; (**B**) Total phenolic compounds. Distinct letters indicate statistical difference between means, according to Tukey’s at a 1% significance level. CM: Culture media (1–4).

Static condition (S) results in higher protein concentration across all evaluated culture media (CM), with values close to 100 µg·mL^−1^ of proteins, except for CM3S (Figure 1A). However, a low influence of the culture medium and agitation factors on TCP content was observed, with the highest value recorded in CM1S (53.81 mg GAE·g^−1^ of extract) and the lowest in CM3S (31.78 mg GAE·g^−1^ of extract) (Figure 1B).

ANOVA indicated that all factors and interactions significantly affected soluble protein content (*p* < 0.05). Glucose showed the most significant effect (*p* = 4.12 × 10^−9^). The interaction between glucose and peptone (*p* = 2.35 × 10^−8^) and between glucose and agitation (*p* = 6.91 × 10^−5^) also exhibited strong significance. Additionally, negative effect estimates indicated that glucose and agitation negatively impacted protein production. The R^2^ value of 0.9934 indicates that the variability in protein production could be explained by the factors and their interactions included in the model (Table 3).

### 3.2. Structural Characterization

#### 3.2.1. Scanning Electron Microscopy (SEM)

**Figure 2:**
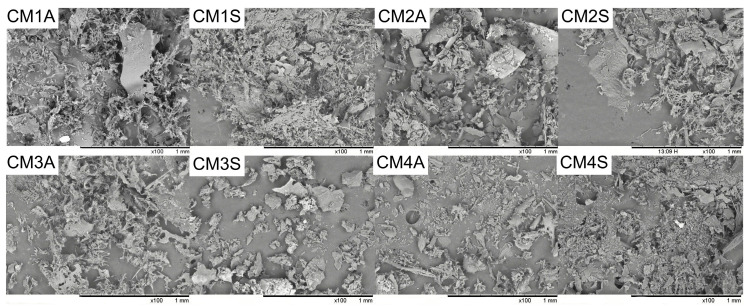
Scanning electron microscopy of protein extracts from *G*. *sichuanense* mycelial biomass cultivated in different submerged conditions. CM: Culture Media (1–4). A: Agitated. S: Static.

The SEM pictures revealed variations in microstructure of the protein extracts depending on the submerged fermentation condition. Most extracts exhibited aggregates, while CM1S and CM3A displayed more fibrillar structures. CM1A and CM2S showed both agglomerates and fibrillar structures (Figure 2).

#### 3.2.2. X-Ray Diffraction (XRD)

**Figure 3:**
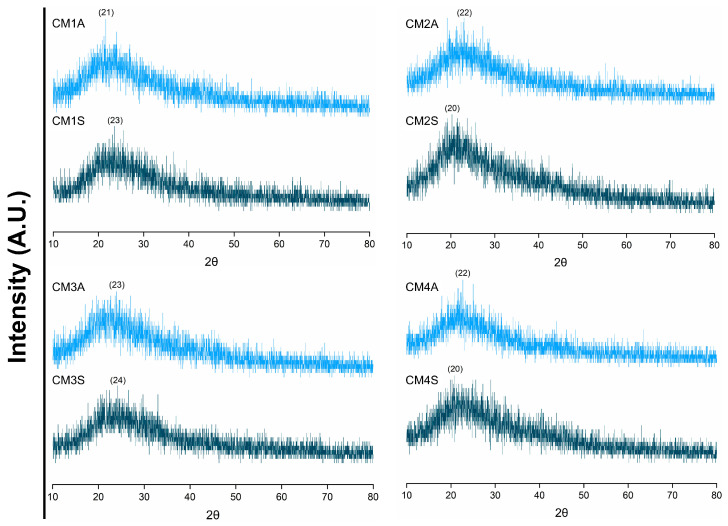
X-ray diffraction patterns of protein extracts from *G*. *sichuanense* mycelial biomass cultivated in different submerged conditions. CM: Culture media (1–4). A: Agitated. S: Static.

XRD analyses demonstrated that the different submerged fermentation conditions had no influence on the crystallinity of the protein extracts. All protein extracts displayed a broad background pattern, typical of amorphous structures, with diffraction peaks at 2θ and values between 20 and 24° (Figure 3).

#### 3.2.3. Attenuated Total Reflectance Fourier-Transform Infrared (ATR-FTIR)

**Figure 4:**
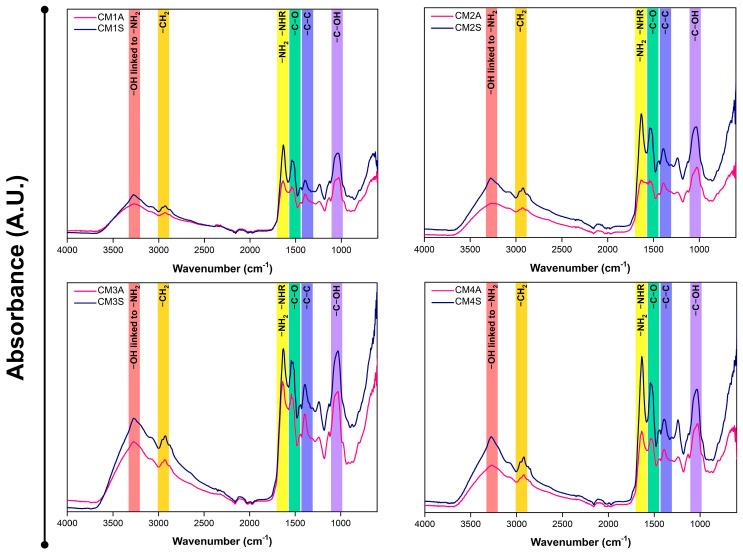
FTIR-ATR spectra of protein extracts from *G*. *sichuanense* mycelial biomass cultivated in different submerged conditions. CM: Culture media (1–4). A: Agitated. S: Static.

FTIR analysis of the protein extracts revealed a high degree of similarity in their absorption profiles, with variations in transmittance dependent on both the culture medium composition and agitation conditions (Figure 4). Protein extracts from non-agitated submerged fermentations displayed reduced transmittance, suggesting a correlation with higher protein content.

**Figure 5:**
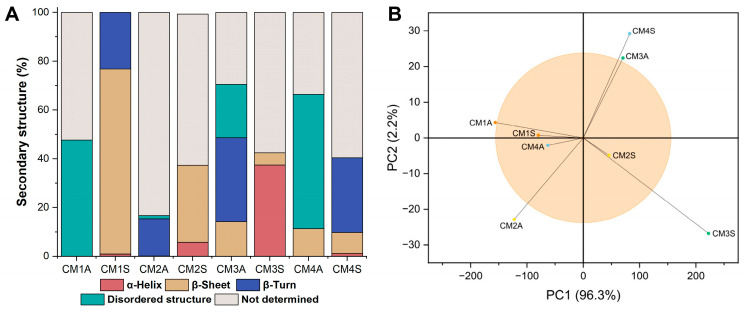
FTIR-ATR data analysis. (**A**) Percentage of secondary structures (**B**) PCA analysis applied to the FTIR-ATR spectra. CM: Culture media (1–4). A: Agitated. S: Static.

The secondary structure of the samples varied considerably depending on the cultivation conditions employed. Most samples exhibited structural conformations that could not be fully determined, except for CM1S, whose structure was completely estimated (Figure 5A). CM1S was composed of β-sheet structures (75.77%), β-turns (23.21%), and α-helices (1.00%). Principal component analysis (PCA) of the FTIR spectra revealed that both agitation conditions and culture medium composition exert a significant influence on the chemical composition of the protein extracts (Figure 5B). The loading analysis highlighted that the region between 1620 and 1640 cm^−1^, corresponding to the protein marker, contributed most to the differentiation among samples.

### 3.3. Antioxidant Activity

**Figure 6:**
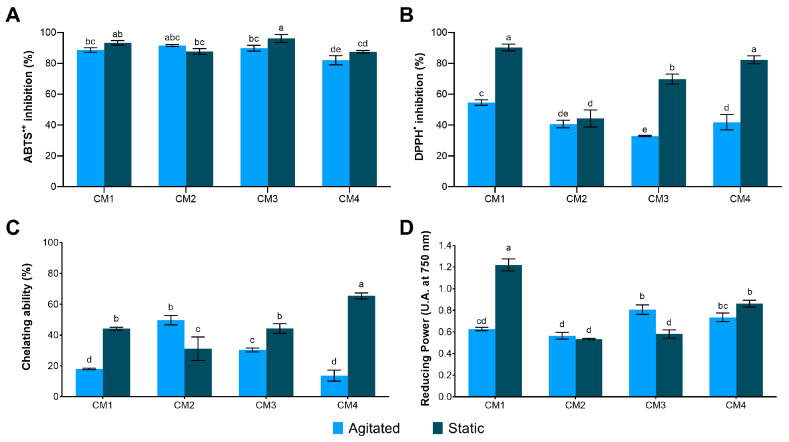
Antioxidant activity in protein extracts from the mycelial biomass of *G*. *sichuanense* cultivated in different submerged conditions. (**A**) ABTS^•+^; (**B**) DPPH^•^; (**C**) Chelating ability; (**D**) Reducing power. Different letters indicate statistically significant differences between means according to Tukey’s test at a 1% significance level. CM: Culture media (1–4).

In vitro antioxidant potential analysis revealed a high capacity for ABTS^•+^ radical scavenging activity in all protein extracts, with values exceeding 80% (Figure 6A). However, DPPH^•^ inhibition was more selective, with only CM1S, CM3S and CM4S displaying significant activity (Figure 6B).

The Fe^2+^ chelating capacity was significantly enhanced in CM4S (65.43 ± 1.97%), compared to the other samples (Figure 6C). In the assessment of Fe^3+^ reducing power, the highest activity was also observed in CM1S (1.220 ± 0.056), confirming its superior antioxidant potential (Figure 6D). The CM1S sample also exhibited the highest capacity to inhibit ABTS^•+^ and DPPH^•^ radicals, as evidenced by the lowest IC_50_ values, requiring lower protein concentrations to inhibit 50% of these radicals (Table 4).

### 3.4. Electrophoretic Profile

**Figure 7:**
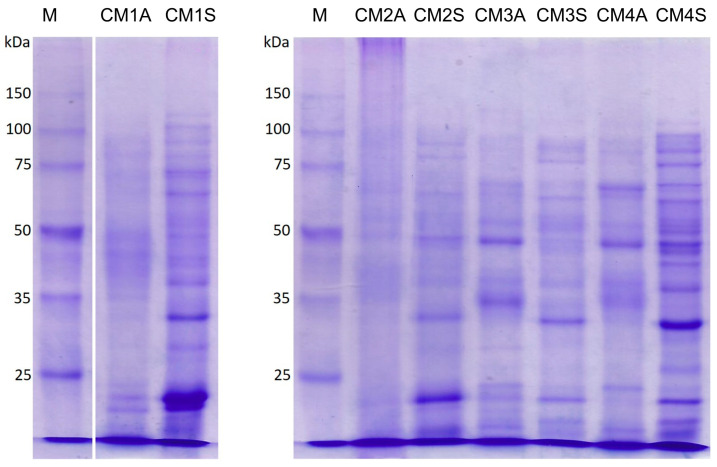
SDS-PAGE of protein extracts from the mycelial biomass of *G*. *sichuanense* grown under different submerged fermentation conditions. M: Molecular weight marker. CM: Culture media (1–4). A: Agitated. S: Static.

Electrophoretic analysis revealed a heterogeneous protein profile among the extracts, with bands raging in molecular weight from ~12 and 125 kDa. The CM1S and CM4S extracts exhibited a more intense and complex band pattern, which is consistent with the higher protein content and enhanced antioxidant activities observed in these extracts (Figure 7), suggesting a correlation between the protein composition and the biological activities.

### 3.5. Cytoprotective Activity

**Figure 8:**
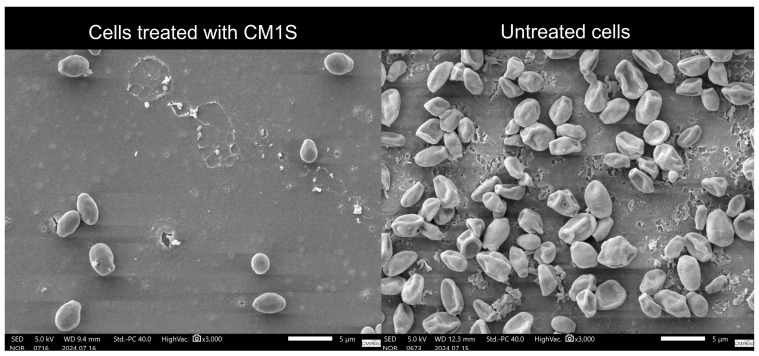
Scanning electron microscopy of *S*. *cerevisiae* cells incubated with H_2_O_2_, untreated and treated CM1S protein extract. The image qualitatively illustrates the morphological changes in yeast cells, without providing quantitative data on cell density.

Scanning electron microscopy (SEM) revealed clear differences in the surface morphology of *S. cerevisiae* cells exposed to H_2_O_2_ in the presence or absence of CM1S (Figure 8). This assay provides a qualitative assessment of cell-surface integrity and supports the interpretation of a cytoprotective effect under oxidative stress. Cells treated with H_2_O_2_ alone exhibited altered morphology, with compressed and distorted cells. In contrast, cells pre-incubated with the CM1S maintained their normal cellular morphology, displaying turgid cells with smooth cell surfaces, indicating a protective effect against oxidative damage.

### 3.6. Bioprocess Parameters from the CM1S

The culture medium 1 under static cultivation (CM1S), which yielded the protein extract with high antioxidant activity and cytoprotective potential, was further investigated to characterize the growth kinetics of *G*. *sichuanense*. Two growth models were fitted to the experimental data to estimate the specific growth rate. Model selection was based on the adjusted R-squared (R^2^*adj*), corrected Akaike Information Criterion (AICc) and root mean square error (RMSE). The Gompertz model best described the growth of *G*. *sichuanense*, as it exhibited lower AICc and RMSE values and higher R^2^*adj* (Table 5).

**Figure 9:**
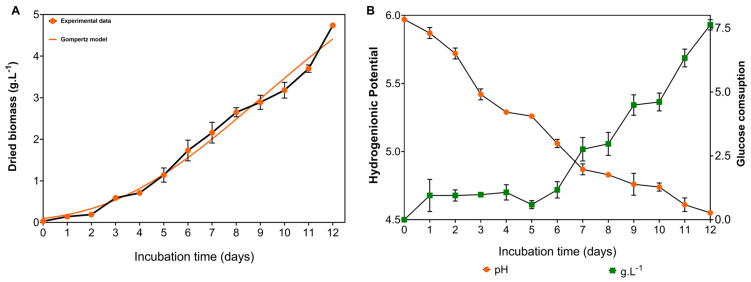
Growth kinetics of *G. sichuanense* cultivated in a medium containing glucose (10 g·L^−1^), soy peptone (2.5 g·L^−1^), and yeast extract (5 g·L^−1^), under non-agitated conditions. (**A**) Biomass production; (**B**) glucose consumption and culture broth pH.

The growth kinetics of *G*. *sichuanense* followed a typical microbial growth curve, comprising a lag phase during the first two days, followed by a well-defined exponential phase. This exponential phase, marked by a high rate of cell division, extended from day 3 until the end of the cultivation period (Figure 9A). Fungal growth was directly associated with significant changes in the culture medium pH and sugar consumption. The progressive drop in pH throughout the cultivation, reaching 4.55 ± 0.01 by the end of the process, along with the sharp increase in sugar consumption starting on day 7 and reaching 76.3% by the end of the experiment, highlights the fungus intense metabolic activity during the exponential growth phase and the overall efficiency of the process (Figure 9B).

**Figure 10:**
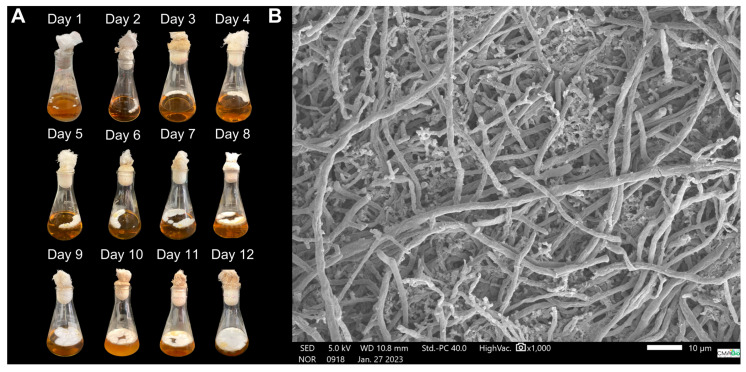
Morphology of the mycelial biomass of *G. sichuanense* CC22 grown in a medium containing glucose (10 g·L^−1^), soy peptone (2.5 g·L^−1^), and yeast extract (5 g·L^−1^), under non-agitated conditions. (**A**) Macromorphology; (**B**) Micromorphology.

The fungal macromorphology demonstrates mycelial dispersion throughout the cultivation period, increasing the contact surface with the liquid medium, facilitating nutrient assimilation, and supporting vigorous fungal growth, eventually covering the entire surface of the culture medium by day 12 (Figure 10A). Microscopy of the biomass on day 12 reveals a mixture of long, tubular hyphae and short, highly branched hyphae, referred to as “staghorn-like hyphae” (Figure 10B).

## 4. Discussion

The soluble protein contents observed in *G*. *sichuanense* are comparable to those in the edible mushroom *Pleurotus ostreatus*, which presented values ranging from 35 and 160 μg/mL at pH levels from 2 to 8 [25]. The variation in soluble protein content between agitation conditions may be linked to the absence of shear forces in non-agitated fermentation. Such mechanical stress can damage the mycelium and promote leakage of intracellular components, a sensitivity previously documented for *G. lucidum* [26,27].

The analyses of protein extracts are often complex due to the presence of phenolic compounds and other substances [28]. Although dialysis steps in protein extraction can effectively remove these compounds, this is not the case when they are complexed with proteins [29], as seems to be the case in this study. However, protein-polyphenol interactions can mask phenolic antioxidant activity. Thus, the observed activity may be attributed to the proteins themselves or to the newly formed complex [30].

In simple systems, such as food matrices, protein–polyphenol complex formation occurs under suitable pH, ionic strength, and temperature conditions, and is primarily mediated by non-covalent interactions, including hydrogen bonding, hydrophobic interactions, electrostatic forces, and van der Waals interactions. These associations can be advantageous, as they often enhance antioxidant activity and enable the development of functional systems such as emulsions, gels, packaging films, and delivery platforms for bioactive compounds [31]. Within this context, the CM1S protein extract emerges as a promising candidate for the intended applications.

Salting-assisted extraction is a well-established technique known for enhancing extraction efficiency while preserving protein functionality. NaCl is commonly used to promote salting-in, where low salt concentrations typically have minimal impact on proteins. The weak interactions formed between charged ions and proteins help prevent aggregation and increase protein solubility. However, this effect can vary depending on factors such as protein concentration, which could explain the variation in the observed microstructures [32,33].

One of the factors that can influence the functional properties of protein materials is their average particle size [34]. Particle size directly affects solubility, and smaller particles typically exhibit higher solubility due to the greater surface area available for hydration [35]. An important factor to consider, as it can impact the industrial application of protein extracts by influencing their techno- and bio-functional properties [36,37].

A study that evaluated different concentrations of NaCl in the extraction of proteins from *Moringa oleifera* seeds exhibited structures resembling those observed in the present work at a concentration of 0.15 M. The authors reported that these structures are suitable for analysis of protein solubility, surface hydrophobicity, and spectroscopy [38].

The diffraction pattern obtained in XRD is commonly reported for amorphous structures [39]. This finding is particularly interesting from an application standpoint, as amorphous solids are more soluble and stable compared to crystalline structures, making them more suitable for pharmaceutical applications [40]. The diffraction peaks, in turn, are characteristic of β-sheet secondary structures, while variations in their intensity suggest alterations in the overall protein conformation [41]. Similar diffraction patterns have been previously reported for whey protein isolate [42] and extracts from *G*. *lucidum* [43], supporting our findings.

The spectral bands observed in FTIR-ATR are characteristic of proteins, aligning with previous findings for whey protein isolates [44]. The presence of bands at 3280, 2920, 1630, 1540, 1400, and 1050 cm^−1^ are attributed, respectively, to stretching vibrations of -OH linked to -NH_2_, the presence of -CH_2_ groups, primary and secondary amide groups of proteins, and -C-O, -C-C, and C-OH bonds, indicative of the protein nature of the extracts.

In *G*. *lucidum*, the infrared spectral region between 1700 and 1500 cm^−1^, attributed to amine bond stretching vibrations, proved highly informative regarding protein content, particularly for the FIP-glu/LZ-8 protein. This spectral region exhibits minimal variations, reflecting the stability of protein content [45]. Peaks near ~1648 cm^−1^ can serve as a reliable biochemical marker indicative of protein content [46].

The CM1S protein extract exhibited secondary structures commonly found in Fungal Immunomodulatory Proteins, including those isolated from *G*. *lucidum* (FIP-glu) [47], which may suggest the presence of this class of proteins in the sample. The high proportion of disordered structures observed in the other samples may reflect the presence of intrinsically disordered protein regions or regions whose structural resolution could not be resolved by the method employed [48]. The percentage composition of secondary structures in each sample may also be influenced by interactions with other molecules, as the formation of protein–phenol complexes has been reported to impact structural stability, potentially enhancing resistance or inducing structural deformations [49].

Previous studies on the mycelial biomass of *G*. *lucidum* for ABTS^•+^ inhibition reported IC_50_ values of 927 µg·mL^−1^ in aqueous extract [50] and a specific activity of 2.47 µg of protein·mL^−1^ [9]. For DPPH^•^ inhibition in *G*. *lucidum* basidiomata, IC_50_ values of 2067.47 µg·mL^−1^ (aqueous extracts) [51], 9000 µg·mL^−1^ (methanolic extract) [52] and 135.4 µg·mL^−1^ (isolated glycopeptide) [53] have been reported. The results obtained in our study, particularly for CM1S, demonstrate a promising antioxidant potential, highlighting the significance of submerged fermentation conditions in the biosynthesis of bioactive compounds.

The high radical scavenging activity may be related to the presence of proteins rich in amino acids such as tyrosine, phenylalanine, tryptophan, cysteine, lysine, arginine, and histidine, which can act as electron and proton donors, neutralizing free radicals and protecting biomolecules against oxidative damage [54]. The DPPH^•^ and ABTS^•+^ methods differ in their mechanisms of action for evaluating antioxidant capacity. The DPPH^•^ radical can be stabilized by accepting either an electron or a hydrogen atom, whereas ABTS^•+^ preferentially reacts with hydrogen atom donors [55,56]. Due to its higher sensitivity and faster reaction kinetics [57], ABTS^•+^ is more suitable for assessing the antioxidant capacity of proteins, which are predominantly hydrophilic molecules [1].

The result in chelating ability assay is comparable to the observed in methanolic extracts of *G*. *tsugae* basidiomata (65% at 10 mg·mL^−1^) [58] and *G*. *lucidum* polysaccharides (70% at 10 mg.mL^−1^) [59], highlighting the antioxidant potential and metal-chelating capacity of *G*. *sichuanense* protein extracts. As for reducing power, ethanolic extract of *G*. *lucidum* basidiomata demonstrated a considerably lower reducing power, requiring concentrations exceeding 160 mg·mL^−1^ to achieve an absorbance of 1.0 unit [60]. Similarly, the methanolic extract of the basidiomata from edible mushroom *Pleurotus eous* displayed an absorbance value of 1.132 at a concentration of 10 mg·mL^−1^ [61].

The ability of the protein extracts to interact with iron ions, especially in their reduction, can be attributed to the presence of amino acids residues with electron-donating groups, such as histidine (nitrogen), glutamate/aspartate (oxygen) and methionine/cysteine (sulfur), with form coordination complexes with metal ions [62]. The chelating capacity of the protein extracts can be attributed to the presence of functional groups like hydroxyl (-OH), thiol (-SH), and carboxyl (-COOH) in their side chains. These groups act as ligands, forming stable complexes with metal ions [63].

Low molecular weight proteins, as observed in CM1S and CM4S, including FIPS (12–15 kDa), are known to play a crucial role in antioxidant activity of *G*. *lucidum* [5,64]. The results obtained for CM1S and E4NA, with a higher intensity of protein bands below 25 kDa, support this hypothesis, reinforcing the idea that the absence of agitation may favor the production of these antioxidant proteins.

Although enzymatic hydrolysis is often employed to enhance the bioavailability and biological activity of peptides, studies with *G*. *lucidum* indicate that this process can reduce antioxidant activity [64]. Thus, the protein extracts from *G*. *sichuanense* emerge as a promising alternative, as they dispense the need for additional enzymatic treatments, avoiding the complexity and costs associated with hydrolysis process. Moreover, their activity under mild processing conditions suggests potential integration into functional ingredients, fermented bioproducts, or protective agents in microbial or cellular systems, broadening their applicability in biotechnological and industrial contexts.

Other studies evaluating the antioxidant potential of proteins from the mycelium and fermented broth of *G*. *lucidum* report the presence of protein bands absent in the basidioma, suggesting that these proteins are produced in response to stress during submerged fermentation [9,64]. Moreover, *G*. *lucidum* strains exhibit significant variation in the intensity and molecular mass of proteins, which may be attributed to pos-translational modifications, proteolysis, genetic variability, cultivations conditions, and stress [65].

The exposure of fungi to heat and oxidative stress conditions can trigger an increase in the production of ROS, acting as signaling molecules for responses to stress [66], thereby stimulating the synthesis of antioxidant proteins and molecules in a cascading reaction [67,68]. This integrated response aims to protect the organism against oxidative stress and ensure its survival. Although no studies were found on the production of Heat-Shock Proteins (HSP) by *G*. *lucidum* under oxidative stress, its synthesis induced by heat stress during a submerged fermentation process has already been reported [69].

Oxidative stress can induce changes in fungal metabolism, leading to the modulation of the expression of numerous proteins [70]. HSPs, for example, are known to be overexpressed in response to variations in temperature, pH, and oxidative stress, playing a crucial role in maintaining cellular homeostasis [71].

**Figure 11:**
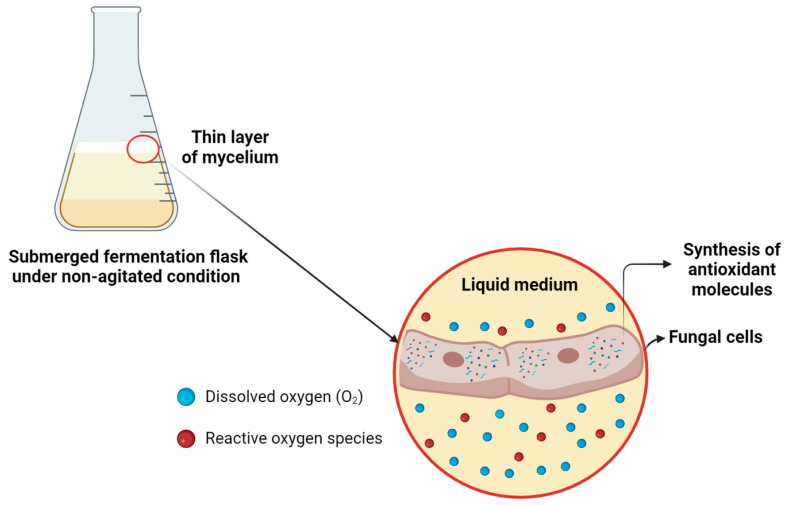
Proposed mechanism of oxidative stress in submerged fermentation under non-agitated conditions of *G*. *sichuanense*. Created in BioRender. Pessoa, V. (2024) https://BioRender.com/k64h913.

Agitation during fermentation acts as an oxygen mass transfer mechanism, maintaining a balance between the amount oxygen supplied and the amount metabolized by the fungus [72]. However, the absence of agitation can restrict oxygen availability, inducing oxidative stress conditions in the culture, as has been previously observed in the submerged fermentation of fungi [73]. Our results indicate that this limitation of oxygen mass transfer may have triggered an adaptative response in the fungus, leading to the overexpression of antioxidant proteins (Figure 11). This suggests that limiting oxygen mass transfer could be a promising strategy for enhancing the production of bioactive compounds with antioxidant activity by *G*. *sichuanense*.

Similar results of cytoprotective activity were reported for a protease inhibitor from *A*. *bisporus* (AbPI), which also exhibited a protective effect on *S*. *cerevisiae* (MTCC 2376) cells treated with 2 mM H_2_O_2_ [18]. These results underscore the potential of CM1S as a cytoprotective agent in various fields, including oncology, where there is an urgent need to mitigate the adverse effects of cancer treatments on healthy cells [74].

The Gompertz model, well-established for its ability to describe microbial growth kinetics under controlled conditions [75] and mushroom biomass accumulation [76], has also proven suitable for modeling biomass accumulation in *G*. *sichuanense* under non-agitated cultivation. In submerged fermentation of *Ganoderma* sp. under agitation at 100 rpm, the Gompertz model was also found to be the best fit for fungal growth, with a specific growth rate of 0.0087 h^−1^ [77], indicating that static cultivation can also be an effective strategy for fungal biomass production.

Despite complete surface colonization of the culture medium by the 12th day, the fungus remained in the exponential phase of growth. This period is particularly suitable for sampling, as it is marked by the active synthesis of primary metabolites, including proteins essential for cell proliferation and development [78]. Therefore, terminating the cultivation at this point is both appropriate and aligned with the objective of maximizing protein yield. The acidification of the culture medium, observed at the end of the process, is a common phenomenon in fungal fermentation and is associated with the production of organic acids from carbohydrate metabolism [79]. The staghorn-like hyphae observed has been described in *G*. *lucidum*, *G*. *carnosum*, *G*. *resinaceum*, *G*. *sinense*, and *G*. *oregonense*, and are more abundant during the development of aerial mycelium [80,81], suggesting an association with protein accumulation, as this differentiated hyphal morphology is typically linked to intensified cell-wall remodeling and increased synthesis of structural and secreted proteins.

Future investigations should focus on characterizing the proteins present in the most active extract. The pharmacological potential of the CM1S sample is currently being assessed in cellular models to confirm its biotechnological relevance. Expanding production to pilot-scale fermentations and evaluating extract stability under different processing conditions should also be pursued to determine its practical feasibility and support its translation into industrial applications.

## 5. Conclusions

Protein extracts obtained from the biomass of *G*. *sichuanense* CC22 were structurally characterized and demonstrated potential for industrial application. The protein extract derived from non-agitated cultivation in a medium composed of 10 g·L^−1^ of glucose, 2.5 g·L^−1^ of soy peptone, and 5 g·L^−1^ of yeast extract, named as CM1S, stood out for its higher concentration of proteins and phenolic compounds, as well as its high antioxidant activity, suggesting the formation of a protein–phenol complex favorable to antioxidant activity. CM1S exhibited a low IC_50_ in ABTS and DPPH assays, high Fe^3+^ reducing power, and cytoprotective activity against H_2_O_2_. From a proteomic perspective, the absence of agitation during cultivation induced the expression of distinct proteins from those observed under agitation, potentially associated with the cascade of reactions resulting from oxidative stress due to the lack of an oxygen mass transfer mechanism. Moreover, the presence of staghorn-like hyphae appears to be associated with biomass containing high protein content, possibly representing a structure more favorable for the accumulation of these molecules. Thus, a cultivation condition that promotes the synthesis of antioxidant proteins by *G*. *sichuanense* is proposed in our work.

## Figures and Tables

**Table 1:** Composition of the culture media in submerged fermentation experiments.

Culture Media (CM)	Concentration (g·L^−1^)		
	Glucose	Soy Peptone	Yeast Extract
No. 1	10	2.5	5
No. 2	20	2.5	5
No. 3	10	5	5
No. 4	20	5	5

**Table 2:** Experimental design matrix for the 2^3^ full factorial design applied to *G*. *sichuanense* submerged fermentation.

Experiment	X_1_ (Glucose)	X_2_ (Peptone)	X_3_ (Agitation)
CM1S	−1	−1	−1
CM2S	1	−1	−1
CM3S	−1	1	−1
CM4S	1	1	−1
CM1A	−1	−1	1
CM2A	1	−1	1
CM3A	−1	1	1
CM4A	1	1	1

**Table 3:** Effects and statistical significance of fermentation variables on protein production by *G*. *sichuanense* in a 2^3^ factorial design.

Factor/Interaction	Estimated Effect	*p*-Value (ANOVA)	Significance
Agitation	−155.14	5.02 × 10^−18^	Highly significant
Peptone	−88.47	1.27 × 10^−2^	Significant
Glucose	1.43	4.12 × 10^9^	Highly significant
Agitation/Peptone	108.47	1.47 × 10^−10^	Highly significant
Glucose/Peptone	85.96	2.35 × 10^−8^	Highly significant
Glucose/Agitation	4.15	6.91 × 10^−5^	Significant
Glucose/Peptone/Agitation	−64.70	1.53 × 10^−5^	Significant

The significance of each factor is categorized as Highly significant (*p* < 0.001), significant (0.001 < *p* < 0.05), or not significant (*p* > 0.05), based on the *p*-value of the ANOVA.

**Table 4:** IC_50_ values of protein extracts for ABTS^•+^ and DPPH^•^ radical scavenging.

Experiment	ABTS^•+^		DPPH^•^	
	IC_50_ (µg of Sample·mL^−1^)	IC_50_ (µg of Protein·mL^−1^)	IC_50_ (µg of Sample·mL^−1^)	IC_50_ (µg of Protein·mL^−1^)
CM1A	1225.0 ± 114.5	42.9 ± 4.0	9681.3 ± 270.5	339.2 ± 9.5
CM1S	507.2 ± 19.4	4.8 ± 0.2	2550.0 ± 96.5	24.0 ± 0.9
CM2A	927.4 ± 10.0	29.6 ± 0.3	ND	ND
CM2S	637.4 ± 11.2	6.0 ± 0.1	ND	ND
CM3A	1405.0 ± 210.6	36.5 ± 5.5	ND	ND
CM3S	1852.0 ± 96.6	29.9 ± 1.6	8134.7 ± 296.8	131.5 ± 4.8
CM4A	1711.3 ± 89.5	32.9 ± 1.7	ND	ND
CM4S	1064.2 ± 79.7	10.1 ± 0.8	6474.3 ± 216.0	61.3 ± 2.0

ND: not determined.

**Table 5:** Mathematical models for the growth kinetics of *G. sichuanense*.

Modelo	Specific Growth Rate (h^−1^)	R^2^*_adj_*	AICc	RMSE
Exponential	0.0084	0.9484	−80.2	0.3327
Gompertz	0.0068	0.9793	−114.4	0.2077

Medium containing glucose (10 g·L^−1^), soy peptone (2.5 g·L^−1^), and yeast extract (5 g·L^−1^) under static conditions.

## Data Availability

The data generated and analyzed in this study are not publicly available as they are part of a broader research project and are subject to privacy and confidentiality restrictions. However, the data may be made available by the corresponding author upon reasonable request, subject to approval by the project’s research team.

## Abbreviations

The following abbreviations are used in this manuscript:

FIP	Fungal Immunomodulatory Protein
HaCaT	Human Aneuploid Keratinocyte Cell Line
B16 cells	Melanoma Cells
Asp	Aspartic acid
Val	Valine
His	Histidine
Cys	Cysteine
Met	Methionine
Se-GL-P	Selenium-Enriched Protein
CAT	Catalase
SOD	Superoxide Dismutase
ROS	Reactive Oxygen Species
INPA	Instituto Nacional de Pesquisas da Amazônia
PDA	Potato Dextrose Agar
CM	Culture Media
A	Agitation condition
S	Static condition
SEM	Scanning Electron Microscopy
XRD	X-Ray Diffraction
ATR-FTIR	Attenuated Total Reflectance Fourier-Transform Infrared Spectroscopy
SDS-PAGE	Sodium Dodecyl Sulfate—Polyacrylamide Gel Electrophoresis
YPD	Yeast Peptone Dextrose
DNS	3,5-dinitrosalicylic acid
ANOVA	Analysis of Variance
PCA	Principal Component Analysis
TCP	Total Phenolic Compounds
AICc	Akaike Information Criterion
RMSE	Root Mean Square Error
HSP	Heat-Shock Proteins
